# Weight-related behaviors and weight loss maintenance: a cross-sectional study in Cyprus

**DOI:** 10.1186/s12889-021-11153-5

**Published:** 2021-06-10

**Authors:** Yiannis Koutras, S. Chrysostomou, K. Giannakou, M. Yannakoulia

**Affiliations:** 1 Department of Life Sciences, School of Sciences, European University Cyprus, 2404 Nicosia, Cyprus; 2 Department of Health Sciences, School of Sciences, European University Cyprus, Nicosia, Cyprus; 3 Department of Nutrition and Dietetics, School of Health Sciences and Education, Harokopio University Athens, Kallithea, Greece

**Keywords:** Obesity, Weight loss maintenance, Eating behavior, Diet

## Abstract

**Background:**

This study examined the differences between maintainers and regainers regarding obesity related eating behaviors. A secondary objective was to develop an eating behavior index predicting the likelihood of successful weight loss maintenance.

**Methods:**

The current cross-sectional evaluation conducted in Cyprus was part of the MedWeight (Greek) study. Eligible for participation were Cypriot (maintainers = 145; regainers = 87) adult men and women who reported being at least overweight (BMI ≥25 kg/m^2^) and experienced an intentional weight loss of ≥10% of their maximum lifetime weight, at least 1 year before participation. Among other assessments, weight-related behaviors were evaluated through Weight-Related Behaviors Index (WRBI).

**Results:**

Statistically significant differences between the two groups were observed regarding meals per day (*P* = 0.008), frequency of eating home cooked meals (*P* = 0.004) and WRBI total score (*P* = 0.022). Results from logistic regression models indicated that the odds of maintaining weight loss increase at 30% (Model 1: *P* < 0.05, Odds ratio 1.306, 1.095–1.556 95% C.I., Model 2: *P* < 0.05, OR 1.308, 1.097–1.560 95% C.I.) and at 38% after adjusting for physical activity (Model 3: *P* < 0.05, OR 1.377, 1.114–1.701 95% C.I..) for each point scored in WRBI total score.

**Conclusions:**

Eating more frequently home cooked meals and less eating away from home meals may be beneficially associated with weight loss maintenance. WRBI seems to be a useful tool when dealing with patients who have previously lost significant weight.

**Supplementary Information:**

The online version contains supplementary material available at 10.1186/s12889-021-11153-5.

## Background

Weight loss maintenance has become the greatest challenge in the management of obesity. Despite the fact that many people manage to lose weight, the percentage of those who succeed in maintaining this loss remains low [1, 2]. There is currently no consensus on the definition of weight loss maintenance in adults. Some studies proposed a definition of intentional weight loss and maintenance for at least 6 months [3], while others proposed a definition of weight loss ≥5% of initial loss and maintenance for at least 1 year [4]. Achieving an intentional loss of ≥10% of maximum body weight and maintaining it for ≥1 year is the most widely used description of successful weight loss maintenance [5, 6], and based on it, ≥20% of at least overweight individuals who had previously lost weight manage in maintaining a significant amount of weight loss [5–9].

Registries of individuals with successful long term weight loss have been developed worldwide to explore factors leading or related to successful maintenance of weight loss [6, 10–13]. Notably, data within these registries include information on socioeconomic background, medical history of individuals as well as data related to individuals’ lifestyle such as sleep, physical activity, and dietary patterns. In particular, higher levels of physical activity, regular monitoring of diet and body weight were the most frequently reported factors [4, 14–17]. In relation to the diet, several studies have examined specific nutrients, foods or dietary schemes as potential factors affecting weight loss maintenance; however, none of these studies has managed to identify a single nutrient, food or even a specific dietary scheme as the most effective [18].

Recent guidelines related to the management of overweight and obesity refer to the importance of incorporating behavioral techniques into various obesity treatment interventions and promote healthy weight-related behaviors [19, 20]. Findings from Weight Control Registries support that reducing energy intake by high frequency of self-weighing and regular meal frequency may promote weight loss maintenance [13]. In addition, it is apparent that individuals who maintain their weight report the use of more behavioral techniques [14, 15]. However, evidence supporting the use of specific weight-related behaviors that promote weight loss maintenance of overweight or obese individuals, who have previously lost significant weight, is scarce [13–15]. Specifically, few behaviors were previously studied mostly on a piecemeal basis rather that in the context of an index that could potentially evaluate their additive, complementary or synergistic effect. In addition, comparisons between maintainers and regainers with significant prior weight loss, could enable researchers to identify important target behaviors for daily practice.

The current study aimed to explore differences in weight-related behaviors between maintainers and regainers enrolled in the MedWeight control registry in Cyprus. Moreover, another aim was to develop a weight-related behavior index predicting the likelihood of weight loss maintenance which could be used as a simple target tool by the Health Care Professionals to monitor weight loss maintenance.

## Methods

### Study population

The present cross-sectional evaluation is based on the data collected from Cypriot participants and it is part of the MedWeight (Greek) study, a registry of weight loss maintainers and regainers [10]. Eligible for participation in the registry were adult men and women aged 18–65 years of Cypriot ethnicity, who reported being at least overweight (Body Mass Index ≥25 kg/m^2^) and experienced an intentional weight loss of ≥10% of their maximum lifetime weight, at least 1 year before participation in the study. Each participant was classified as “maintainer” if his/her current weight was ≤90% of his/her maximum weight or “regainer” if his/her current weight was ≥95% of his/her maximum weight. Participants who had a current weight between 91 and 94% of their maximum weight were excluded to avoid overlapping between the two groups.

This study was conducted according to the guidelines laid down in the Declaration of Helsinki and the study protocol was approved by the National Bioethics Committee. The recruitment procedure held for 2 years (2/2018–2/2020) and it was communicated through press releases, advertisements in tv, radio and social media. All eligible participants signed the consent form prior to participation in the study and were then advised to access a web-based platform (http://medweight.hua.gr) to fill in a series of questionnaires. Specifically, volunteers were asked to report socio-demographic status such as marital status (single, married/cohabitating, divorced, widowed, then coded for married/cohabitating or not), occupational status (employed or not) and years of education. Eligible volunteers were asked to report physical/personal characteristics such as sex, age, weight, height, BMI, maximum weight, maximum BMI, initial loss, maintenance loss and duration of maintenance.

### Assessment of dietary intake

Two telephone 24-h dietary recalls were conducted for each participant in order to assess dietary intake [21]. The recalls were performed by two well-trained dietitians within the period of 10 days for each participant, with weekdays and weekends proportionately represented among participants. Using the multiple-pass method, dietitians asked for all foods and beverages consumed the previous day [22, 23]. Dietitians were blinded regarding the participant’s maintenance status. All data were analyzed in terms of total daily energy intake by using the dietary analysis software SNPRO Nutrition Software (Cheapsoft Softwares, 2017).

### Assessment of weight-related behaviors and development of the weight-related behaviors index (WRBI)

The frequency of weight-related behaviors was also assessed. We selected specific behaviors that have been previously reported to be associated either with weight loss and/or weight loss maintenance [24–31]. Specifically, in the web-based platform, participants were asked to report about the frequency of eating out, eating with others, eating breakfast (rarely/never, 1–3 times per month, 3–6 times per week, daily, more than twice a day), the number of eating episodes per day (1–3 eating episodes, 4–5 eating episodes or ≥ 6 eating episodes per day), the number of main meals per day (1–3 main meals per day) and eating visible fat or meat skin (almost all, part of or none). Moreover, other questions related to food supplements (yes or no), eating rate (very fast, fast, medium, slow, very slow), food preparation (yes or no), person responsible for food preparation (mostly you or mostly others) and eating home-cooked meals (almost never, sometimes, often, almost always) were also included.

The weight-related behaviors responses were collectively evaluated through the Weight-Related Behaviors Index (WRBI), a simple to understand and easy to use index developed specifically for the current study. This index consisted of 10 variables as listed above; 9 of these variables were related to eating behaviors and 1 variable was related to the frequency of self-weight measurement. In particular, the variables used for the development of the index were: eating out, eating with others frequency, eating breakfast frequency, eating episodes per day, main meals per day, eating rate, time spent on food preparation, responsible for food preparation, eating home cooked meals frequency, and self-weighing frequency. Behaviors related to food supplements and eating visible fat or meat skin were excluded in the final index due to lack of strong supporting evidence from previous studies regarding their association with weight loss and/or weight loss maintenance. Based on the results of differences between the responses of the two groups, the variables were then coded to dichotomous types after previously developed nutritional indexes were taken into account [32–35]. Each variable was scored with 0 or 1 (0 indicates less healthy behavior and 1 indicates more healthy behavior). The scoring system allowed the development of more distinct categories for each variable of the final index. As an example, for the eating out frequency, rarely/never and 1–3 times per month were coded to 1 and 3–6 times per week, daily or more than twice a day were coded to 0. The score range is 0–10: the higher the score is the more the individual is engaged in a behavior that it is expected to promote weight loss maintenance.

### Assessment of physical activity

The short version of the International Physical Activity Questionnaire (IPAQ) validated for the Greek population was used to assess physical activity through our web-based platform [36]. Participants were asked to report high, intermediate, and low intensity activities lasting ≥10 min, as well as sedentary activity and time spent during these activities on a weekly basis.

### Statistics

Using Q-Q plots we explored normality of distribution of data. Normally distributed values were presented as means and standard deviation (SD), non-normally distributed values as medians and interquartile range (IQR) and data from categorical variables as frequencies (in percentage). We explored differences between maintenance status in participants’ characteristics using independent t test or Mann Whitney rank tests, depending on the normality of the data, and chi-square tests for categorical variables. Cronbach’s alpha was used as a measure of internal consistency. Differences between maintainers and regainers were tested by logistic regression models for categorical variables (results were expressed as odds ratio [95% confidence interval]).

Logistic regression models were performed using maintenance status as a dependent variable (1 = maintainer, 0 = regainer) and WRBI total score as independent variables: Model 1 was adjusted for age, sex, education level (years of education) and marital status (married or not); Model 2 was additionally adjusted for energy intake; Model 3 was additionally adjusted for physical activity (IPAQ total Met-minutes per week). Analysis of the receiver operating characteristic (ROC) was conducted to determine the optimal cut-off value of the WRBI that differentiates maintainers from regainers. Crucial point was defined by the largest distance from the diagonal line of the ROC curve. Data analysis was carried out using SPSS Statistics 22.0; a *P*-value of 0·05 was considered statistically significant.

## Results

Table 1 presents the characteristics of all the participants enrolled in the study, based on their weight maintenance status. In particular, 232 men and women enrolled in this study of which 145 were maintainers and 87 regainers. Concerning the total sample, 52.2% were women, 29.7% married and most of them employed (60.2%). Regainers were older than maintainers (37.3 ± 14.4 years vs 33.0 ± 12.2 years, *P* < 0.05) and they also had less years of education (13.3 ± 4.2 years vs 15.1 ± 3.2 years, *P* < 0.05). Moreover, initial weight loss was significantly higher among the maintainers compared to regainers (26.7 ± 16.0 kg vs 15.4 ± 7.6 kg, *P* < 0.05) and maintainers reported maintaining this loss for over 3 years. Although maintainers reported a lower weight and BMI, their max weight, max BMI and initial weight loss was higher than regainers (*P* < 0.05 for all comparisons).

**Table 1:** Participants’ characteristics (*N* = 232)

	Total (*n* = 232)	Maintainers (*n* = 145)	Regainers (*n* = 87)	*P* value
Sex (% female)	52.20	51.70	52.90	0.893
Marital status (% married)	29.70	33.10	41.40	0.396
Employment status (% employed)	60.20	69.40	72.90	0.524
Age (years)	34.61 (13.21)	33.00 (12.19)	37.29 (14.43)	**0.022**
Weight (kg)	82.41 (18.86)	77.61 (17.49)	90.43 (18.43)	**0.001**
BMI (kg/m2)	28.67 (5.48)	26.90 (4.86)	31.60 (5.19)	**0.001**
Max weight (kg)	96.13 (22.10)	98.38 (23.61)	92.38 (18.83)	**0.045**
Max BMI (kg/m^2^)	33.41 6.13)	34.08 (6.45)	32.29 (5.40)	**0.031**
Initial loss (kg)	22.46 (14.52)	26.72 (15.96)	15.37 (7.62)	**0.001**
Weight loss maintained (%)		12.44 (12.66)		
Maintaining years		3.37 (3.14)		
Education years	14.45 (3.69)	15.11 (3.18)	13.31 (4.20)	**0.002**

*Abbreviations*: *ΒΜΙ* Body mass index, Statistically significant results are denoted in bold, Values are presented as mean (standard deviation) for quantitative variables and relative frequencies for qualitative variables

For the development of WRBI index, the responses of the participants were grouped into two categories for each item of the index in order to simplify the final index scoring (Table 2). The Cronbach’s alpha score was considered acceptable (Cronbach’s alpha = 0.68). Results after the development of the WRBI index showed statistically significant differences between the responses of two groups regarding the following weight-related behaviors: eating episodes per day (*P* = 0.008) and frequency of eating home cooked meals (*P* = 0.004). There was a trend towards significance regarding frequency of eating out (*P* = 0.058). Although no statistically significant differences were observed regarding the other behaviors, their inclusion in the WRBI index was considered critical to provide a more comprehensive assessment of nutritional behavior, as described in other indexes or scores when assessing diet and other lifestyle factors [32, 33, 37]. Regarding the WRBI total score, statistically significant results were observed between the two groups (*P* = 0.022). Notably, results from logistic regression models indicated that for every point scored in WRBI total score the odds of maintaining weight loss increase at 30% (Model 1: *P* < 0.05, Odds ratio 1.306, 1.095–1.556 95% Confidence interval., Model 2: *P* < 0.05, OR 1.308, 95% CI: 1.097–1.560) and increase to 38% after adjusting for physical activity (Model 3: *P* < 0.05, OR 1.377, 95% CI: 1.114–1.701). Additional adjustment for baseline BMI did not change the results.

**Table 2:** Weight-related eating behaviors index (WRBI) scoring system and frequencies of responses

	Score (0/1)	Maintainers (%)	Regainers (%)	***P*** value
**Eating out frequency**				
rarely/never	1	60	45.5	0.058
1–3 times per month				
3–6 per week	0	40	54.5	
daily				
more than twice a day				
**Eating with others frequency**				
rarely/never	0	71.3	62.1	0.203
1–3 times per month				
3–6 per week				
daily	1	28.7	37.9	
more than twice a day				
**Eating breakfast frequency**				
rarely/never	0	38.3	45.5	0.343
1–3 times per month				
3–6 per week				
daily	1	61.7	54.5	
**Eating episodes per day**				
1–3 eating episodes	0	74.8	90.9	**0.008**
4–5 eating episodes				
≥ 6 eating episodes	1	25.2	9.1	
**Main meals per day**				
1	0	34.8	42.4	0.307
2				
3	1	65.2	57.6	
**Eating rate**				
very fast	0	38.3	47	0.252
fast				
medium	1	61.7	53	
slow				
very slow				
**Time spent on food preparation**				
≥ 1 h	1	87.8	83.3	0.399
< 1 h	0	12.2	16.7	
**Responsible for food preparation**				
mostly you	1	60.9	60.6	0.972
mostly others	0	39.1	39.4	
**Eating home cooked meals frequency**				
almost never	0	47.8	69.7	**0.004**
sometimes				
often				
almost always	1	52.2	30.3	
**Weighing frequency**				
daily	1	50.4	36.6	0.063
2–6 times per week				
weekly				
1–3 times per month	0	49.6	63.4	
a few times per year or never				
**WRBI Max Total Score**		5 (3–7)	4 (1–6)	**0.022**

Values are presented as median (IQR) or relative frequencies. Statistically significant results are denoted in bold

The performance of the WRBI was useful in discriminating maintainers among individuals, with an area under the receiver operating characteristic curve (AUC) of 0.589 (*P* = 0.023, 95% CI: 0.514–0.664.). The best cut-off value for the WRBI was ≥1.50 (Sens/1-Spec: 0.779/0.724) (Supplemental Figure 1).

## Discussion

The current study aimed to examine the association between eating behaviors and weight loss maintenance among adults who had previously lost weight and maintained it or regained it. The main findings indicate that maintaining weight loss is associated with having at least 6 eating episodes per day and almost always eating home cooked meals. Hence, the WRBI total score was positively associated with weight loss maintenance, indicating that the WRBI index could be a useful tool for monitoring weigh loss maintenance and/or as a guide for relevant nutrition counseling. Health care professionals need more practical tools to address weight management success. It was therefore considered wise to develop an index incorporating weight-related behavior that could potentially affect weight loss maintenance. A more holistic approach which incorporates interactions of eating behaviours, such as the WRBI index, may produce many benefits of clinical research and practice than any other single variable. To our knowledge this is the first study to produce such a weight-related behaviours index. Previous efforts regarding the development of similar tools were mostly based on foods or nutrients [38–40], and not on behaviors.

In relation to specific behaviors, our results indicated that maintainers reported having more eating episodes per day than regainers. This is in line with the results of a previous study examining self-reported eating frequency of main meals and snacks consumed per day in weight loss maintainers, normal weight, and overweight individuals. Although there were no differences regarding participants’ main meals per day, maintainers and normal weight individuals consumed more snacks than overweight individuals [41]. However, results from the National Weight Control Registry in the USA, highlighted that maintenance of weight loss could also be achieved by having fewer eating episodes, a behaviour reported mostly by older people [42]. However, findings regarding the effect of meal frequency in weight loss maintenance seem to be conflicting and a possible reason could be the fact that different definitions of “a main meal” or “a snack” were used in various studies. For the purposes of the current study, we decided to choose having ≥6 eating episodes daily as the healthy behavior, due to reasons related with the inclusion of comparison groups and distinct definition of main meal/snack reported by previous study [41].

Although there is a great heterogeneity across studies examining the relationship between meal patterns and body weight mainly due to the method of assessment of main meals and snacks, most of them indicate a negative association between higher frequency of home cooked meals and body weight, in accordance with our study. As an example, in a cross sectional study of 11,936 participants, it was found that eating home cooked meals more frequently is associated with lower adiposity and better diet quality [43]. Previous studies indicated that eating home cooked meals could help individuals monitor their food and energy intake and that self-monitoring of food and energy intake was positively related to weight maintenance status [44, 45]. In any case, evidence across the above studies is a result of the cross-sectional nature and therefore, causation cannot be inferred. Further clarification is required through other prospective studies.

The strength of this study is that, to the best of our knowledge, the population used has not yet been examined related to weight loss maintenance. In addition, the population was consisted of both maintainers and regainers which allowed for direct comparisons of obesity related eating behaviors, as well as young volunteers, whereas in most relevant studies participants were middle-aged [15, 16, 46, 47]. Moreover, the use of two 24-h recalls as assessment tools allowed a more detailed dietary assessment. This study has also some limitations. In particular, the observational nature of this study indicates associations, but no causation can be drawn. Another limitation of the study is the fact that all data derived from questionnaires (e.g., weight measurement and dietary recalls) were self-reported, a fact which could potentially lead to misreporting and information bias. Yet, this design of contacting has been previously reported that might enhance response rates and improve sample representations, while maintain unbiased outcomes [48, 49]. Moreover, the tendency to underreport is a commonly observed behavior in studies of individuals with overweight or obesity, weight loss maintainers do not seem to underreport in a greater degree than individuals with overweight or obesity [50]. Also, no information was collected regarding the type of the weight loss program used by the participants, however it seems that despite the dietary method used for weight loss the results of weight maintenance do not appear to differ [51]. The small number of participants and the fact that they were not equally distributed in the two groups, resulting in a group of maintainers twice the size of the regainers’ group, are another limitation. However, it is unlikely that this inequality could have influenced our results as previously reported [52]. Lastly, the AUC demonstrated fair to poor discriminatory ability most likely due to the small number of participants. Therefore, further studies are required to assess the discriminative ability of the Weight-Related Behaviors Index in predicting weight regain vs. maintenance.

## Conclusions

Adoption of healthy eating behaviors, such as eating more frequent home cooked meals and less eating away from home meals may influence weight loss maintenance. A Weight-Related Behaviors Index was designed to incorporate specific obesity-related eating behaviors previously shown to influence long term weight loss. The use of WRBI index by the Health Care Professionals could be either an assessment or a target tool to support individuals with overweight or obesity to maintain their weight after significant weight loss. Studies exploring the usability of this index in other setting or populations groups and its ability to discriminate between successful weight losers or maintainers and not are required in the upcoming future.

## Supplementary Information

**Additional file 1: Figure S1.** Receiver operating characteristic curve of the Weight-related Behaviors Index for weight loss maintenance success of the study participants.

## Data Availability

The datasets generated during and analyzed during the current study are not publicly available due to due to confidentiality but are available from the corresponding author on reasonable request.

## Abbreviations

AUCs: Areas under the receiver operating characteristic curves
BMI: Body Mass Index
WRBI: Weight-Related Behaviors Index
IPAQ: International Physical Activity Questionnaire
SD: Standard Deviation
IQR: Interquartile Range
OR: Odds Ratio
CI: Confidence interval
ROC: Receiver Operating Characteristic
