# Supplementary material for: Weight-related behaviors and weight loss maintenance: a cross-sectional study in Cyprus
Source: BMC Public Health. 2021 Jun 10;21:1115. doi: 10.1186/s12889-021-11153-5 (PMC8194136; doi:10.1186/s12889-021-11153-5)

Figure 1. Receiver operating characteristic curve of the Weight-related Behaviors Index for weight loss maintenance success of the study participants.


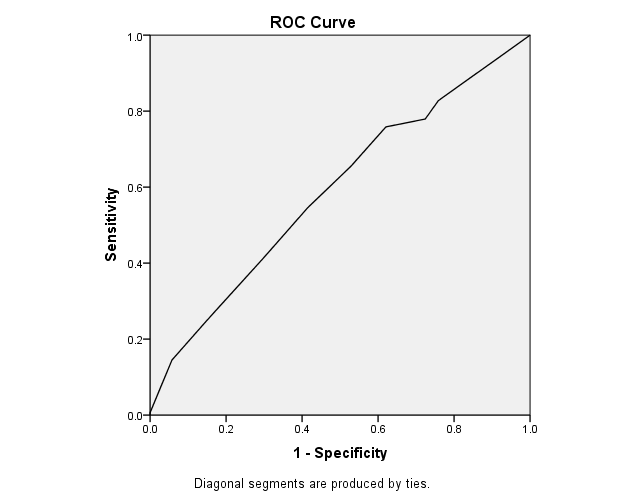

Supplement: Supplementary file 1 — Additional file 1: Figure S1. Receiver operating characteristic curve of the Weight-related Behaviors Index for weight loss maintenance success of the study participants. [file 12889_2021_11153_MOESM1_ESM.docx]
